# Breast Cancer Cell Cultures on Electrospun Poly(ε-Caprolactone) as a Potential Tool for Preclinical Studies on Anticancer Treatments

**DOI:** 10.3390/bioengineering8010001

**Published:** 2020-12-22

**Authors:** Bianca Bazzolo, Elisabetta Sieni, Annj Zamuner, Martina Roso, Teresa Russo, Antonio Gloria, Monica Dettin, Maria Teresa Conconi

**Affiliations:** 1 Department of Pharmaceutical and Pharmacological Sciences, University of Padua, 35131 Padova, Italy; bianca.bazzolo@studenti.unipd.it (B.B.); mariateresa.conconi@unipd.it (M.T.C.); 2 Department of Theoretical and Applied Sciences, University of Insubria, via Dunant, 3, 21100 Varese, Italy; 3 Department of Industrial Engineering, University of Padova, via Marzolo, 9, 35131 Padova, Italy; annj.zamuner@unipd.it (A.Z.); martina.roso@unipd.it (M.R.); monica.dettin@unipd.it (M.D.); 4 Institute of Polymers, Composites and Biomaterials, National Research Council of Italy, V.le J.F. Kennedy 54-Mostra d’Oltremare Pad. 20, 80125 Naples, Italy; teresa.russo@cnr.it (T.R.); antonio.gloria@cnr.it (A.G.)

**Keywords:** breast cancer, polycaprolactone, in vitro models, electroporation, mechanical analysis

## Abstract

During anticancer drug development, most compounds selected by in vitro screening are ineffective in in vivo studies and clinical trials due to the unreliability of two-dimensional (2D) in vitro cultures that are unable to mimic the cancer microenvironment. Herein, HCC1954 cell cultures on electrospun polycaprolactone (PCL) were characterized by morphological analysis, cell viability assays, histochemical staining, immunofluorescence, and RT-PCR. Our data showed that electrospun PCL allows the in vitro formation of cultures characterized by mucopolysaccharide production and increased cancer stem cell population. Moreover, PCL-based cultures were less sensitive to doxorubicin and electroporation/bleomycin than those grown on polystyrene plates. Collectively, our data indicate that PCL-based cultures may be promising tools for preclinical studies.

## 1. Introduction

Traditionally, two-dimensional (2D) cultures, where cells are grown as monolayers on flat plastic plates or in suspension, have been widely used to study cancer development and screen antiproliferative drugs. Despite being easy to handle, cost-effective, and fast to grow, the correlation of results from 2D cultures to in vivo scenarios has been questioned. Indeed, when compared to native environment, 2D cultures almost completely lack cell–matrix interactions. Furthermore, the diffusion-limited distribution of oxygen, nutrients, metabolites, and signaling molecules as well as certain signaling pathways involved in cell growth, metabolism, and differentiation are lost [1]. On the other hand, in vivo models are expensive, time-consuming, and present genetic, metabolic, and ontogenetic profiles different to humans [2]. In this context, three-dimensional (3D) cultures have shown to be promising tools for preclinical studies during drug development. Indeed, they resemble the tumor microenvironment, by promoting both cell–cell and cell–matrix interactions, presenting gene and protein expression patterns like those observed in solid tumors, and forming drug gradients as in the in vivo scenario [3,4]. So far, several 3D cultures have been obtained by using both natural, such as Matrigel and hyaluronan, and synthetic, such as poly(vinyl alcohol), scaffolds [5,6].

Poly (є-caprolactone) (PCL), a synthetic polymer belonging to the aliphatic polyester family, is biocompatible, since its degradation product, caproic acid, is non-toxic and can be easily eliminated from the body [7]. It is characterized by good rheological and viscoelastic properties, compatibility with a wide range of other polymers, and good processability [8]. All these features have led to PCL approval by the Food and Drug Administration for human applications, such as suture material and drug delivery systems [9]. PCL has been also extensively studied for biomedical applications to obtain both porous and fibrous scaffolds for bone, cartilage, blood vessels, skin, nerve, tendon, and ligament replacements [7,10].

Among the fabrication technologies applied to process PCL into 3D scaffolds, electrospinning is a cost-effective and simple process that allows the formation of polymer fibers with sub-micron diameter, mimicking the native extracellular matrix (ECM) microstructures, especially the high-ordered collagen microfibrils [11]. Thus, thanks to their porosity, electrospun fiber meshes provide a natural 3D tissue-like structure enabling oxygen and nutrient gradients [11,12]. Various electrospun PCL fibrous scaffolds have been already evaluated to obtain 3D in vitro breast cancer models useful for drug screening and reducing the number of animals in cancer research [3,7,11,13,14,15,16]. Collectively, these scaffolds supported adhesion and growth of various breast cancer cell lines and, interestingly, increased stem cell population [13,14,15]. However, ECM production by cancer cells cultured on electrospun PCL has not been investigated yet. Furthermore, despite some reports that evaluate the response of these cultures to well-known anticancer drugs (i.e., doxorubicin) [14], their reliability for ElectroPoration (EP) studies has not been verified. 

EP is defined as the transient loss of semi-permeability of cell membranes subjected to the voltage pulses, thus leading to “ion leakage, escape of metabolites, and increased uptake by cells of drugs, molecular probes, and DNA” [17,18,19]. Nowadays, EP is a well-established technique for cell transfection, facilitating the transport of different molecules across the plasma membrane and it is currently applied in many different fields. One application of EP is gene electrotransfer (GET), a non-viral method to deliver DNA molecules into cells by means of electric pulses. Interestingly, this approach has been also used to introduce immunomodulatory cytokines (e.g., IL-12) into cancer cells. This technique, called electroimmunotherapy, is applied in ongoing Phase II clinical trials to treat melanoma, carcinoma, and lymphoma [20].

Electrochemotherapy (ECT) is defined as “the local potentiation, by means of permeabilizing electric pulses, of the antitumor activity of a non-permeant (or a low-permeant) anticancer drug possessing a high intrinsic cytotoxicity” [21]. This therapy uses a sequence of voltage pulses applied between a couple of electrodes to generate a local electric field able to permeabilize the cell membrane. In practice, some aqueous pores are formed on the cell membrane and the delivery of some molecules is improved. Besides membrane permeabilization of cancer cells, electric pulses induce a transient reduction of blood flow, leading to a delay in drug elimination from the tumor (so-called vascular lock). ECT exerts also cytotoxic effects on the tumor endothelial cells, thus preventing the neovascularization and a rapid reorganization of the tumor vasculature. Moreover, the massive cell necrosis and tumor antigen shedding stimulates the natural immune response. Collectively, all the above-mentioned EP-mediated effects enhance the effectiveness of anticancer drugs [21,22,23].

ECT is currently used in clinics as a safe and valuable treatment of cutaneous and subcutaneous metastases, malignant melanoma, basal cell carcinoma, head and neck, squamous cell carcinoma, adenocarcinoma of the breast and salivary gland, hypernephroma, Kaposi sarcoma, transitional cell carcinoma of the bladder, and recurrence of breast cancer [22,24,25,26,27]. Starting from these evidences, a lot of effort has been devoted to apply ECT to sarcomas, bone metastasis, and liver and pancreatic cancers [22,28,29,30,31]. However, since the efficiency of ECT depends on the tissue environment that modulates the local electric field intensity and drug uptake, novel 3D in vitro models are needed to obtain reliable results from preclinical studies [32,33,34,35].

**Figure 1:**
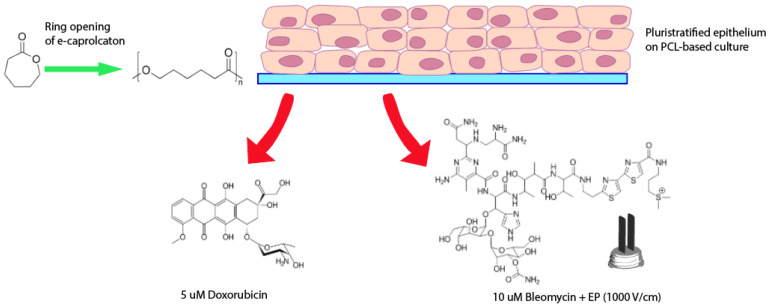
Schematic overview of the experiment set-up.

Herein, electrospun PCL was proposed to support breast cancer HCC1954 (infiltrating ductal carcinoma) cell adhesion and growth, as well as matrix deposition. Cell cultures were characterized by means of morphological analysis, cell viability assays, histochemical staining, immunofluorescence, and mRNA expression analysis. In addition, the responses of breast cancer cells to anticancer drugs and EP were evaluated. Figure 1 gives a schematic overview of the experiment set-up.

## 2. Materials and Methods 

### 2.1. Materials

PCL (MW 60 kDa), sodium cacodylate, sucrose, acetonitrile, propidium iodide (PI), Hoechst 33342 (HOE), hexafluoroisopropanol (HFIP), doxorubicin, and bovine serum albumin (BSA) were obtained from Sigma Aldrich (Steinheim, Germany), and ethanol from VWR Chemicals Prolab (Fontenay-sous-Bois, France). Bleomycin was from Abcam (Cambridge, UK).

The HCC1954 cell line was provided by ATTC (Guernsey, UK). Fetal bovine serum (FBS), culture media, and supplements were obtained from Corning (Mediatech Inc., Manassas, VA, USA). The PrestoBlue™ Cell Viability Reagent and secondary antibody goat anti-mouse Alexa 488 were purchased by Thermo Fisher Scientific (Eugene, OR, USA), whereas paraformaldehyde, Masson’s trichrome, and Alcian blue staining kits by BioOptica (Milan, Italy). The TriReagent and the qPCRBIO SyGreen 1-Step Go Lo-ROX were purchased from PCRBIOSYSTEMS (London, UK), and Gel Red Nucleic Acid staining from Biotium (Hayward, CA, USA). Anti-CD44 antibody was provided by AbD Serotech (Kidlington, UK), and Fluoroshield with DAPI was from Vector Laboratories (Peterborough, UK). Tissue-Tek^®^ OCT was purchased from Thermo Fisher Scientific (Waltham, MA, USA).

### 2.2. Preparation of Scaffolds

PCL-based scaffolds were prepared as follows: a 13% wt/wt PCL solution in HFIP was electrospun using these parameters: metallic static support, 7 cm × 8 cm; pump flow, 0.2 mL/h for 3 h; distance between the collector and the needle, 22 cm; voltage, 20 kV; air coaxial flow, 0.2 bar; environment humidity, 69%; temperature, 22.6 °C, syringe, 2.5 mL; needle, 27 G. The thickness of the electrospun scaffolds was 0.254 ± 0.026 mm (Digimatic micrometer, Mitutoyo Corporation, Kawasaki, Japan). To determine fiber diameters and pore size, one hundred fibers were measured on SEM micrographs by using ImageJ software (NIH National Institutes of Health, Bethesda, MD, USA, https://imagej.nih.gov/ij/).

### 2.3. Tensile Tests

Tensile tests were performed following a previously described procedure [36]. In brief, electronspun PCL meshes were immersed at 37 °C in physiological solution [36]. The tests were carried out on wet specimens with a total length of 20.0 mm, a thickness (t) of 0.08 mm, and a width (w) of 6.0 mm. The gage length (e.g., grip-to-grip distance) (l_0_) was 10 mm. All the tests were performed at a rate of 10 mm/min using an INSTRON 5566 testing machine (Norwood, MA, USA). The engineering stress (σ) was calculated as follows:

(1) $$\sigma ={\frac{F}{A}}_{0}$$

where F and A_0_ represent the force measured by the load cell and the original cross-sectional area of the specimen (t × w), respectively.

The engineering strain (ε) was calculated as the ratio between the elongation (Δl) and the gage length (l_0_):

(2) $$\varepsilon =\frac{∆l}{l_{0}}$$

Tensile modulus, maximum stress, and maximum strain were evaluated and reported as mean value ± standard deviation (SD).

### 2.4. Cell Cultures

HCC1954 cells (derived from human infiltrating ductal carcinoma cells, primary tumor) were cultured in RPMI supplemented with 1% penicillin/streptomycin, 1% L-glutamine, and 10% fetal bovine serum (FBS) at 37 °C and 5% CO_2_ (SteriCult CO_2_ incubator, Thermo Electron Corporation). PCL-based scaffolds were put in either 24-well or 96-well cell suspension culture plates and irradiated with UV light for 1 h in laminar flow hood. Cells (2 × 10^5^ in 24-well plates, 2 × 10^4^ in 96-well plates) were seeded on scaffolds previously hydrated with culture medium for about 30 min. The culture medium was changed every day. At 1, 3, and 7 days from seeding, cell viability was assessed by using the PrestoBlue™ Cell Viability Reagent according to the manufacturer’s instruction. Briefly, at each time point, 10% PrestoBlue™ was added to each well and incubated for 30 min at 37 °C. Then, media were removed, transferred in another plate, and fluorescence (excitation 560 nm emission 590 nm) was read using the Victor^3^ 1420 Multilabel Counter (PerkinElmer, Waltham, MA, USA). Fluorescence values derived from blank, containing PrestoBlue and medium, were subtracted from those cell cultures referred to above. HCC1954 cells cultured on tissue culture polystyrene plates (TCP) were taken as control.

### 2.5. Scanning Electron Microscopy

Scanning Electron Microscope (SEM) analysis was carried out on dried electrospun PCL and cultures grown on PCL-based scaffolds. At 7 days from seeding, cultures were fixed with 3% glutaraldehyde in sodium cacodylate buffer 0.1 M pH 7.2 for 3 h at room temperature (RT). Samples were dehydrated with ascending alcohol solutions (30%, 50%, 70%, 80%, and 90%) for 30 min each and left overnight in 95% ethanol at room temperature (RT). The following day, samples were put in absolute ethanol and subjected to critical point drying. Samples were transferred in a specimen pressure chamber together with liquid CO_2_ at a temperature ranging from 0 to 3 °C and a pressure of 50 bar. Then, they were heated at 40 °C (pressure 80–90 bar) and the CO_2_ critical point was reached at 31 °C and 75 bar. Finally, gold sputtered samples were analyzed by SEM (Cambridge Stereoscan 440 SEM, Cambridge, UK). 

### 2.6. Histological Analysis

At 7 days from seeding, PCL-based cultures were washed in phosphate buffer (PBS) 1× twice and then fixed with 4% paraformaldehyde at 4 °C overnight. The fixed samples were washed and placed in sucrose aqueous solution at increasing concentration (5%, 10%, and 20%) for 1 hour each at 4 °C and left in 30% sucrose at 4 °C overnight. Samples were then embedded in Tissue-Tek^®^ OCT and cut with Feather microtome blades S35 using a Leica cryostat CM 1850 UV. Eight µm sections were collected on Thermo Scientific Superfrost Plus slides, and the freezing medium was removed by washing with phosphate buffer. Alcian blue and Masson’s trichrome staining were carried out by using commercially available kits, according to the manufacturer’s instructions. 

### 2.7. Reverse Transcription Polymerase Chain Reaction (RT-PCR)

At 7 days from seeding, total RNA was extracted using TriReagent according to the manufacturer’s instructions and quantified using NANODROP 2000 (Thermo Scientific, Carlsbad, CA, USA). Primers’ sequences are reported in Table 1. GAPDH was chosen as the housekeeping gene. RT-PCR was carried out through the qPCRBIO SyGreen 1-Step Go Lo-ROX according to the manufacturer’s protocol and using total RNA at a concentration of 75 ng/reaction for each sample. The thermal cycling program consisted of 50 °C for 10 min (reverse transcription), 95 °C for 2 min (DNA-polymerase activation), 40 two-step cycles of 95 °C for 5 s (denaturation), and 62 °C for 25 s (annealing and elongation). The procedure was carried out using the C1000 Touch thermal cycler (Bio Rad, Hercules, CA, USA). The PCR products were separated by 1% agarose gel electrophoresis and visualized by Gel Red Nucleic Acid staining at 1:10,000. The images of the gel were captured with Gel Doc^TM^ Imager (Bio-Rad) and analyzed with Image Lab software (Bio-Rad). To obtain a semiquantitative assessment of gene expression, normalized ratios were obtained by comparing the integrated density values for target genes with those of GAPDH. Then, results were expressed as a percentage with respect to the cultures grown on tissue-culture polystyrene plates considered as 100%.

### 2.8. Immunofluorescence

The inactivation of non-specific binding sites was achieved by incubation with 3% BSA in phosphate buffer (PBS) for 2 h at room temperature (RT). Slides were incubated overnight at 4 °C with the primary non-conjugate mouse monoclonal antibodies anti-CD44 diluted 1:100 in PBS-BSA 1.5%. Then, the samples were incubated for 30 min at RT with secondary antibody goat anti-mouse Alexa 488 diluted 1:200 in 1.5% BSA. Slides were mounted with Fluoroshield with DAPI mounting medium. Negative controls were stained by omitting the primary antibody.

### 2.9. Sphere-Forming Assay

Cells were detached from cell cultures grown on polystyrene plates and 7-day PCL-based cultures. Then, the cells (4 × 10^3^/cm^2^) were seeded into each well of low attachment 24-well plates and cultured with sphere formation medium composed of 450 mL MammoCult Basal Medium (Stemcell technologies, Vancouver, BC, Canada) supplemented with 50 mL MammoCult proliferation Supplement, 1 mL Heparin Solution (final concentration of 4 μg/mL), and 2.5 mL Hydrocortisone (final concentration of 0.48 μg/mL). At 7 days from seeding, cultures were observed by using a phase contrast microscope Nikon T-s (Shinagawa, Tokyo, Japan) and photographed at 40× magnification. The number and sphere diameters were determined using ImageJ tools [37].

### 2.10. Cellular Response to Doxorubicin

HCC1954 cells (2 × 10^4^) were seeded on PCL-based scaffolds previously put on each well of a 96-well plate and cultured for 7 days. Doxorubicin at the final concentration of 5 μM was added in 100 μL of culture medium and, after 24 and 48 h exposure periods, cell viability was determined by PrestoBlue assay. Results were expressed as percentage from the corresponding non-treated cultures. 

### 2.11. Cellular Response to Bleomycin and Electroporation

HCC1954 cells (4 × 10^5^) were seeded in 8-well chamber slides and cultured, as reported in Section 2.4, for 7 days. 

The Electroporation (EP) of the cell cultures on tissue-culture polystyrene and PCL-based scaffolds was performed using the voltage pulse generator EPS-01 (Igea S.p.A, Carpi, Italy). The voltage pulses were applied using a stainless electrode (two rectangular plates with sides 10 and 30 mm long and a gap of 7 mm). Each sample was treated by means of 8 rectangular voltage pulses at 5 kHz (pulse length 100 μs, period 200 μs) with a suitable amplitude [24,38,39]. The pulse amplitude was varied between 140 and 910 V to generate an electric field with intensity from 400 to 1300 V/cm to determine the electroporation curve in adherent cell cultures and PCL-based ones. EP was carried out in culture medium (RPMI) whose electrical conductivity was measured by means of Hanna Instrument electrical conductivity meter HI8883 (Hanna Instrument, Padova, Italy) calibrated at 20 °C using a standard solution, Crison conductivity standard 9710 (1413 µScm^−1^ at 25 °C). 

To verify cell permeabilization, 5 µL PI (1 mg/mL) and 5 µL HOE (1 mg/mL) were added to each well before and after EP, respectively [39,40,41,42]. Then, PI (excitation 538 nm, emission around 619 nm) and HOE (excitation 352 nm, emission around 455 nm) were visualized by using the fluorescence inverted microscope Leica DI4000 (objective 20 × 0.35 DRY, camera DFC300FXR2-078921405). Both blue and red fluorescence images were superposed to brightfield images through the software LAS AF Lite. The red (PI) and blue (HOE) images were used to evaluate the percentage of the electroporated cells as a function of the electric field intensity. The stained cells were counted in the images taken for each sample (at least 5 images per well). The percentage of red cells was evaluated for each electric field intensity applied. The experiments were repeated three times. Furthermore, at 72 h from EP, cell viability was determined by Presto Blue assay, as previously reported. 

Immediately after addition of 10 µM bleomycin in 250 µL culture medium, EP was carried out at 1000 V/cm electric field intensity. After 10 min from EP, medium was removed and substituted with fresh ones (bleomycin-free). After 24 h, cell viability was determined. 

### 2.12. Statistical Analysis 

Data, obtained from at least three experiments, were expressed as mean ± standard deviation. The difference between groups was evaluated using analysis of variance (ANOVA) and Student’s t-test.

## 3. Results

### 3.1. Morphological and Mechanical Features of Electrospun PCL 

**Figure 2:**
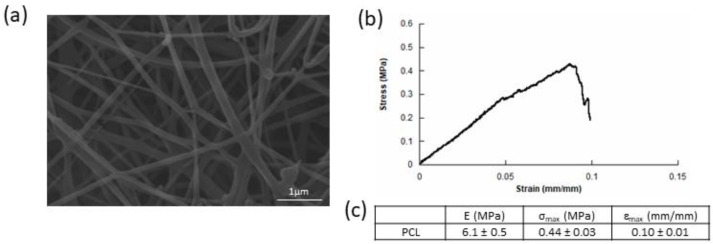
(**a**) Scanning Electron Microscope (SEM) micrographs of dry electrospun PCL without cells. (**b**) Stress-strain curve for electrospun PCL meshes. (**c**) Tensile tests on electrospun PCL meshes: modulus (E), maximum stress (σ_max_), and maximum strain (ε_max_) reported as mean value ± standard deviation (SD).

Figure 2a shows SEM micrographs of electrospun PCL. Scaffolds were composed of a dense network of fibers whose average diameters were 0.22 ± 0.08 µm and pore size 2.10 ± 0.60 µm. Figure 2b reports a typical stress-strain curve obtained from tensile tests on PCL meshes. The results showed a stress-strain curve with an initial linear region followed by a change of the slope, indicating mechanical weakening and progressive local fiber failures, until a maximum stress value was reached. Afterwards, the first macroscopic signs of damage were detected due to fibers’ splitting. Values of tensile modulus (E), maximum stress (σ_max_), and maximum strain (ε_max_) were measured and are reported in Figure 2c. A tensile modulus of 6.1 ± 0.5 MPa, a maximum stress of 0.44 ± 0.03 MPa, and a maximum strain of 0.10 ± 0.01 mm/mm were found for PCL meshes. Such results are consistent with those already reported in a previous work (i.e., 6.3 ± 0.5 MPa, 0.42 ± 0.03 MPa, and 0.11 ± 0.01 mm/mm) [36].

### 3.2. Cell Cultures on Electrospun PCL 

**Figure 3:**
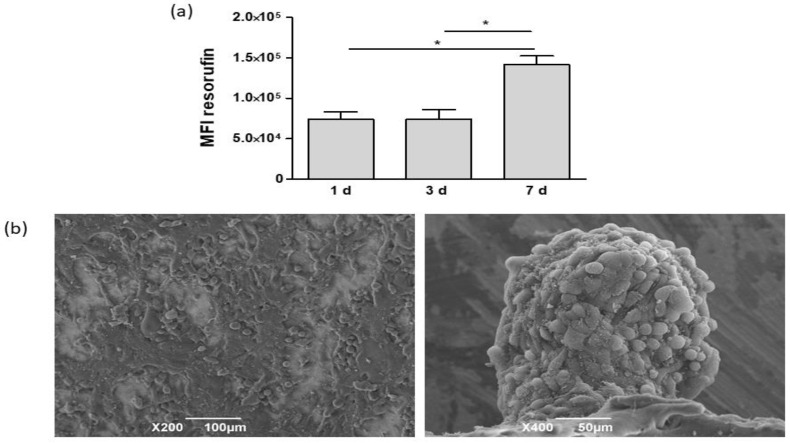
(**a**) Cell viability at various time points from seeding. MFI: mean fluorescence intensity. * *p* < 0.05 vs. corresponding cultures grown on PCL scaffolds. (**b**) SEM micrographs of HCC1954 cell cultures on PCL at 7 days from seeding. Cells completely covered electrospun PCL (left panel, bar: 100 µm) and formed spheroids (right panel, bar: 50 µm).

Cell growth on biomaterials was evaluated at 1, 3, and 7 days from seeding through formation of resofurin, whose mean fluorescence intensity (MFI) correlates with the number of viable cells (Figure 3a). No differences in MFI were detected between 1 and 3 days, whereas at 7 days, cell viability significantly increased. Cell cultures, grown on tissue culture plates, were considered the control group. At 1 day, MFI values were significantly higher than those detected in PCL-based cultures. Nevertheless, at 3 days, cells on tissue culture plates detached because they did not stratify in this culture condition (data not shown). Starting from these data, further experiments were carried out on cultures grown on PCL-based scaffolds at 7 days from seeding. Morphological analysis, carried out by SEM, showed that the scaffold surface was completely covered by HCC1954 cells (Figure 3b). PCL cultures were coated by polygonal shaped cells that were randomly orientated and stratified. Moreover, smaller cells organized themselves to form spheroids laying on the layer generated by the previous cells. 

**Figure 4:**
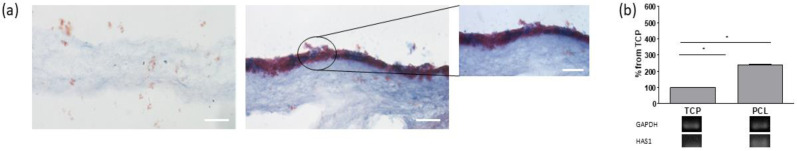
(**a**) Alcian blue staining of cultures grown on PCL at 7 days from seeding. Image on the left shows PCL scaffold without cells, whereas images on the right report PCL-based cultures. Mucopolysaccharides stain blue, and cytoplasm red. Bars: 100 and 50 µm. (**b**) RT-PCR analysis of HAS1 of cultures grown on tissue-culture polystyrene (TCP), and PCL scaffolds at 7 days from seeding. Quantification of transcript levels was carried out by densitometric analysis using ImageLab software. Data were reported as the percentage from TCP taken as 100. * *p* < 0.05 vs. TCP cultures; Student’s t test.

To evaluate the production of ECM components, histochemical staining and gene expression analysis were carried out. The histological analysis highlighted that cells formed a multi-layered coating, but no cells were detected inside the PCL scaffolds (Figure 4a). Alcian blue staining revealed the production of mucopolysaccharides. Positive blue areas were visible in intercellular spaces, and a diffuse staining pervaded all the thickness of biomaterials. Consistent with histological evidence, RT-PCR detected significant increases in transcript levels of HAS1, an enzyme involved in hyaluronic acid synthesis, in PCL-based cultures with respect to that determined in cultures grown on tissue culture polystyrene (TCP) plates (Figure 4b). 

**Figure 5:**
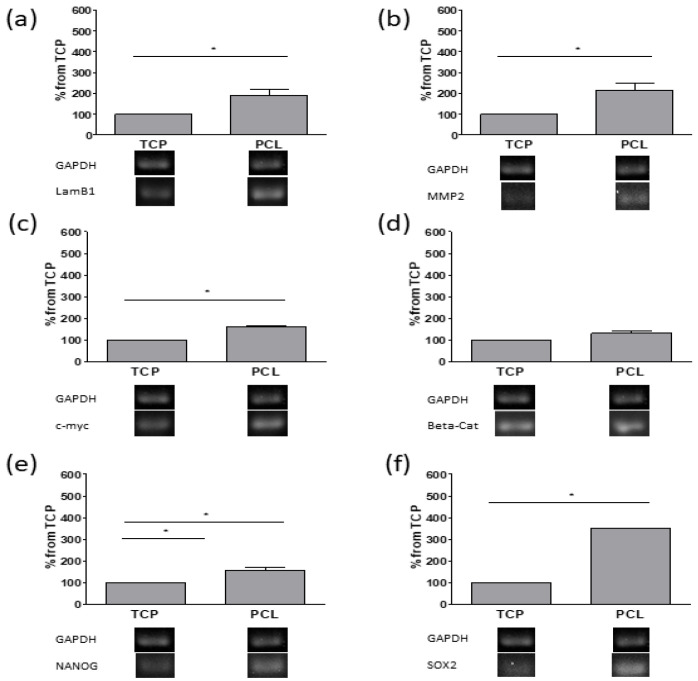
RT-PCR analysis of LamB1 (**a**), MMP2 (**b**), c-myc (**c**), β-cat (**d**), NANOG (**e**), and SOX2 (**f**) of cultures grown on tissue-culture polystyrene (TCP), and PCL scaffolds at 7 days from seeding. Quantification of transcript levels was carried out by densitometric analysis using ImageLab software. Data were reported as percentage from TCP taken as 100. * *p* < 0.05 vs. TCP cultures; Student’s *t* test.

**Figure A1:**
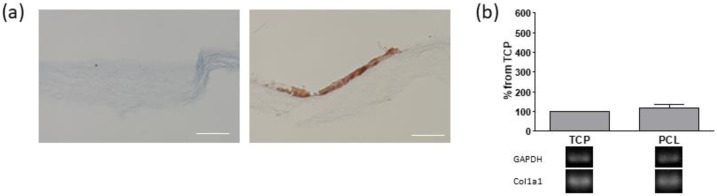
(**a**) Masson’s trichrome staining of cultures grown on PCL at 7 days from seeding. Image on the left shows PCL scaffold without cells, whereas image on the right reports PCL-based cultures. Collagen stains: blue, cytoplasm: red, and nuclei: black. Bars: 100 µm. (**b**) RT-PCR analysis of Col1a1 of cultures grown on tissue-culture polystyrene (TCP), and PCL scaffolds at 7 days from seeding. Quantification of transcript levels was carried out by densitometric analysis using ImageLab software. Data were reported as a percentage from TCP taken as 100. Student’s t test.

On the other hand, no collagen-positive areas were detected by Masson’s trichrome staining and no significant increases in Col1a1 transcripts were detected in PCL-based cultures (see Appendix A, Figure A1). However, RT-PCR revealed significant increases in mRNAs of laminin B1 (LamB1), a component of the basal lamina, and matrix metalloproteinase 2 (MMP2), involved in tissue remodeling (Figure 5). Furthermore, transcript levels of c-myc, NANOG, and SOX2, involved in cell proliferation, stem cell renewal, and maintenance, were also enhanced in PCL-based cultures with respect to 2D adherent cultures. No variations in β-cat transcript levels were detected.

**Figure 6:**
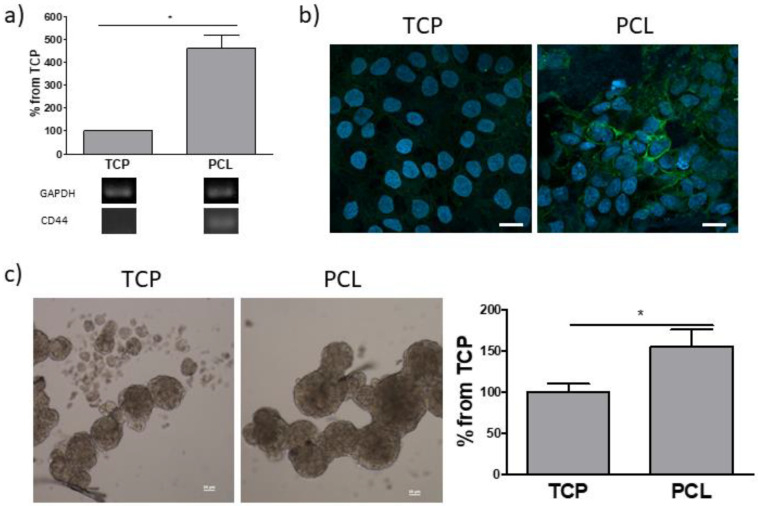
(**a**) RT-PCR analysis of CD44 of cultures grown on tissue-culture polystyrene (TCP), and PCL at 7 days from seeding. Quantification of transcript levels was carried out by densitometric analysis using ImageLab software. Data are reported as the percentage from TCP cultures taken as 100. * *p* < 0.05 vs. control cultures; Student’s t test. (**b**) Immunofluorescence of CD44: nuclei and CD44-positive areas are blue and green, respectively. Bars: 20 µm. (**c**) Mammosphere forming assay. Left panel: representative phase contrast micrographs (bars: 50 µm). Right panel: mammosphere quantification. Data are reported as the percentage from TCP cultures taken as 100. * *p* < 0.05 vs. control cultures; Student’s t test.

Then, the expression of CD44, a transmembrane glycoprotein acting as a hyaluronan receptor, was evaluated (Figure 6a). Both RT-PCR and immunofluorescence demonstrated higher CD44 levels in on PCL-based cultures than that observed in adherent cell cultures. Since CD44 is also a surface marker of cancer stem cells (CSCs) [43], the sphere forming assay, which functionally assesses the presence of a stem cell population and the capability of reinitiate tumor formation, was carried out (Figure 6b). A significantly higher number of spheres arose from cells previously cultured for 7 days in PCL with respect to those derived from cells grown on tissue-grade plastic. 

### 3.3. Responses of PCL-Based Cultures to Anticancer Drugs and EP

**Figure 7:**
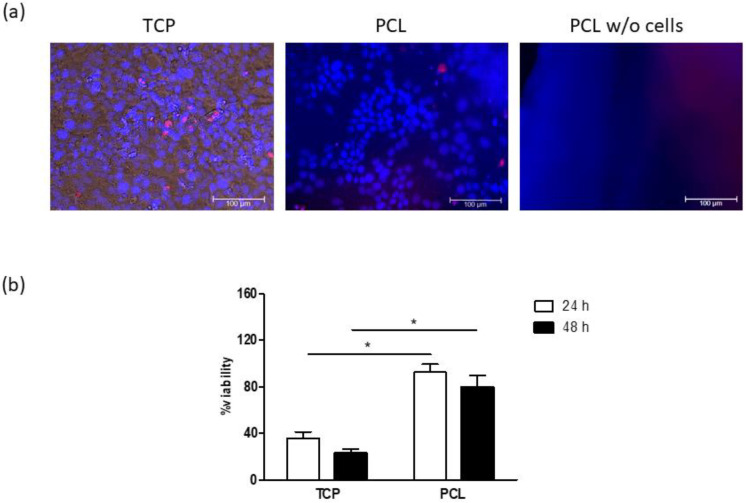
(**a**) Representative images of cultures at 15 min of doxorubicin treatment: nuclei and doxorubicin are blue and red, respectively. Bars: 100 µm. (**b**) Cell viability after 24 and 48 h exposure periods to 5 µM doxorubicin. Results are reported as percentage from the corresponding non-treated cultures. * *p* < 0.05 vs. cultures grown on tissue-culture polystyrene (TCP); Student’s *t* test.

To evaluate cultures on PCL-based scaffolds as tools for preclinical studies, a comparison with 2D adherent cell cultures was carried out through two drugs, namely doxorubicin and bleomycin, widely used in anticancer therapy. Cultures grown on TCP and PCL-based scaffolds were treated with 5 µM doxorubicin for 24 and 48 h and cell viability was determined by a resazurin-based assay (Figure 7). After a 15 min exposure period, doxorubicin signal was well visible into several cells grown on TCP, whereas drug uptake was limited to a few cells in PCL-based cultures, as revealed by fluorescence microscopy (Figure 7a). Furthermore, no signal was observed when doxorubicin was added to electrospun PCL without cells. At both 24 and 48 h, cell viability was significantly lower in cultures grown on TCP than that detected in cultures on PCL (Figure 7b). 

**Figure 8:**
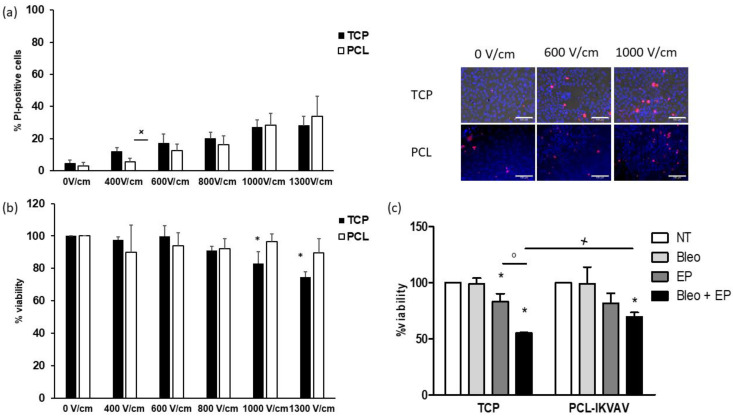
Effects of EP and EP/bleomycin on cell cultures. (**a**) Right panel: PI uptake. Results are expressed as the percentage of PI-positive cells. ^x^ *p* < 0.05 vs. corresponding cultures on tissue-culture polystyrene (TCP); Student’s t test. Left panel: representative micrographs of cultures stained with PI (red) and HOE (blue). Bars: 100 µm. (**b**) Cell viability at 72 h from EP. Results are expressed as percentage from corresponding non-treated cultures (0 V/cm) taken as 100. * *p* < 0.05 vs. corresponding cultures grown on TCP; Student’s t test. (**c**) Cell viability at 24 h from EP/bleomycin (Bleo). Results are expressed as the percentage from corresponding non-treated cultures (NT). * *p* < 0.05 vs. corresponding untreated cultures; ° *p* < 0.05 vs. corresponding electroporated cultures; ^x^ *p* < 0.05 vs. corresponding Bleo + EP-treated cultures.

EP was carried out on adherent cell cultures on TCP and PCL-based cultures by applying an increasing electric field intensity. In non-treated cultures (0 V/cm), only electrode was inserted, and no electric field was applied. Cell membrane permeabilization was assessed through PI staining, whereas cell viability was determined at 72 h from EP (Figure 8a,b). In both cell cultures, the percentage of PI-positive cells was progressively enhanced by increasing electric fields and reached about 30% at high voltages. No differences were observed between the two culture models, except at 400 V/cm, where significantly higher values were detected in cultures grown on TCP. However, in PCL-based cultures, cell viability was not affected by the electric field application, whereas significant decreases were detected in culture grown on TCP at 1000 and 1300 V/cm. Adherent cells and PCL-based cultures were treated with 10 µM bleomycin and electroporated by applying 8 pulses, 100 µs long, delivered at 5 kHz and using a voltage amplitude of 700 V (electric field intensity of 1000 V/cm). After a 24 h incubation period, cell viability was assessed (Figure 8c). As expected, bleomycin alone showed no effects in both cultures. On the other hand, EP significantly decreased cell viability in cultures grown on TCP, but not in PCL-based ones. Cytotoxic effects of EP/bleomycin were significantly higher in cells grown on TCP compared to those detected in PCL-based cultures. 

## 4. Discussion

During anticancer drug development, the majority of drug candidates selected by in vitro screening are ineffective in in vivo studies and clinical trials because of the unreliability of 2D in vitro cultures that are unable to mimic the cancer microenvironment. In this work, electrospun PCL was evaluated to obtain breast-cancer like in vitro models. 

Electrospinning gave rise to a network of fibers whose diameters (about 0.2 µm) were comparable to the ones of collagen, thus mimicking the fibrous part of ECM. Moreover, mechanical analysis highlighted that the electrospun PCL exhibited suitable properties with a tensile modulus of about 6 MPa.

PCL-based scaffolds supported cell growth, as demonstrated by the increases in cell viability detected from 1 to 7 days. Cells formed on electrospun PCL formed a pluristratified coating resembling an epithelial coverage, and they did not penetrate the scaffold due to pore dimensions (about 2 µm) of the biomaterial. Thus, the lack of collagen was an expected result, because collagen fibers are abundant in the connective tissues but not in the epithelial one, where the amount of ECM is small. However, cells on electrospun PCL produce mucopolysaccharides, as demonstrated by Alcian blue staining. This evidence was confirmed by the higher transcript levels of HAS1, an enzyme involved in hyaluronic acid (HA) production [44], detected in PCL-based cultures, with respect to cells grown on tissue culture-grade plastic. 

HA is highly expressed in several tumors, where it has been related with poor patient outcome [45]. The interaction of HA with its cell surface receptors, such as CD44, activates the transduction of intracellular signals involved in cell differentiation, survival, proliferation, migration, angiogenesis, invasiveness, and resistance to therapeutic molecules. It has also been shown that HA is involved in the maintenance of cancer stem cells (CSCs): in the presence of HA, tumor cells express stem-like markers, such as Oct4, Sox2, NANOG, and also ATP-binding cassette transporters [46].

Interestingly, SEM analysis revealed two cell types on PCL-based cultures: cells randomly oriented formed a pluristratified coating, whereas smaller cells organized themselves into spheroids, due to CSCs that are usually present in a small percentage in cancer cell lines. 

Our results indicate that electrospun PCL enhances the CSCs population, in agreement with other articles on PCL scaffolds having various pore and fiber dimensions [13,14]. Herein, an increase in both some stem cell markers and in sphere-forming ability was detected in PCL-based cultures with respect to those grown on polystyrene plates. Transcriptional and expression levels of CD44, overexpressed in breast tumor cells [47], were enhanced in PCL-based cultures. CD44 is the main ligand of HA, and their interaction leads to the activation of different cell signaling pathways (among them, Ras, MAPK, and PI3K) that enhance cell adhesion, migration, and proliferation. Moreover, CD44/HA is related to the expression of multidrug resistance gene (P-glycoprotein) and the activation of anti-apoptotic pathways, which promotes drug resistance and survival [43]. Furthermore, transcript levels of c-myc, NANOG, and SOX2 increased in PCL-based cultures. The gene c-myc is over-expressed in breast cancer [48] and SOX2 and NANOG are critical regulators of self-renewal, as well as pluripotency of embryonic stem cells, and are CSC markers. In breast cancer, SOX2 increases cell proliferation, colony formation, and cell metastasis [49]. NANOG contributes to carcinogenesis via activating and preserving CSCs properties by regulating some genes or pathways, such as Stat3/Snail EMT, in many carcinomas, including breast cancer [50,51].

To evaluate whether PCL-based cultures could be suitable for preclinical studies of anticancer drugs, cultures were treated with doxorubicin, commonly used in clinics for the treatment of breast cancer [52]. Cells grown on PCL-based scaffolds were less sensitive to drug cytotoxic effects than conventional adherent cultures. As suggested by the low drug uptake after a 15 min incubation period, this result may be due to the different drug diffusion pattern that, in turn, is affected by ECM deposition, cell stratification, and heterogeneity detected in PCL-based cultures.

Furthermore, the proposed in vitro model was also evaluated as a tool to study EP and its applications. Although PCL has already been used to obtain a breast cancer in vitro model [7,13], the potential application of PCL-based cultures for EP studies has not been explored. EP induces cell permeabilization by application of an external electric field and its outcome depends on various parameters, such as pulse features (amplitude, duration, pulse number, and repetition rate), membrane composition, surrounding media, the orientation of cells in the tissue, and temperature [53]. Nowadays, novel in vitro approaches are required to assess the complex responses to EP since the knowledge on the electric field distribution in tissues is almost lacking. Indeed, the theoretical models so far developed are able to describe the EP phenomena for the simple cases of cells in suspension or in monolayer [54]. Herein, in both adherent and PCL-based cultures, maximum values (about 30%) of cell permeabilization were obtained at high electric field intensities (1000 and 1300 V/cm). However, only in 2D cultures were decreases in cell viability were detected. 

Bleomycin is one of the most widely used drugs in ECT for the treatment of several tumors, such as cutaneous cancers [55]. Since it is a non-permeant molecule internalized by endocytosis, its uptake is limited by the number of carrier proteins and by the speed of proteins withdrawn from the membrane. EP allows bleomycin to diffuse almost freely inside the cells. Thus, a few hundred molecules are sufficient to kill the cell and bleomycin cytotoxicity is increased up to ~8000-fold without side effects [23,56]. 

Our data showed that the cytotoxic effects of EP/bleomycin were more severe in adherent cell cultures than those grown on PCL scaffolds. Interestingly, at 1000 V/cm, cell permeabilization was comparable in the two culture systems, as demonstrated by PI uptake. Thus, the differences in cell sensitivity may be due to the increase in CSCs population detected in PCL-based cultures. Indeed, it is well-known that CSCs exhibit resistance to toxic agents and reinitiate tumor formation following primary therapy [47].

## 5. Conclusions

Collectively, our data showed that electrospun PCL allows the in vitro formation of cultures characterized by mucopolysaccharides production and increased CSCs population. Our results and the evidence demonstrating that cell-ECM interactions [57] boost tumor cells’ stemness led us to hypothesize that the enhancement of CSCs population may be due to HA through increases of CD44 expression. PCL-based cultures were less sensitive to doxorubicin and EP/bleomycin than adherent cell cultures. This chemoresistance could be related to increases in CSCs. Moreover, the presence of ECM affected cell organization and drug diffusion profile, thus contributing to the decrease in sensitivity to anticancer treatments. Further studies will be necessary to better characterize the proposed in vitro model for drug screening applications.

## Figures and Tables

**Table 1:** Primers for polymerase chain reaction (PCR) amplification. F: forward; R: reverse.

Gene	Primer Sequence
GAPDH	F- TCTTCCAGGAGCGAGATC R- CAGAGATGATGACCCTTTTG
Hyaluronic Acid synthase 1 (HAS1)	F- TGCGATACTGGGTAGCCTTC R- GGTTGTACCAGGCCTCAAGA
Collagen 1a1 (Col1a1)	F- GACTGGTGAGACCTGCGTGT R- TTGTCCTTGGGGTTCTTGCT
Laminin B1 (LamB1)	F- GCGAGAATCCCAGTTCAAGG R- GGGGTGTTCCACAGGTCATT
Matrix Metalloproteinase 2 (MMP2)	F- CGACCGCGACAAGAAGTATG R- TGTTGCCCAGGAAAGTGAAG
c-myc	F- CTCCACACATCAGCACAACTA R- TGTCCAACTTGACCCTCTTG
β-Catenin (β-cat)	F- CTTCACCTGACAGATCCAAGTC R- CCTTCCATCCCTTCCTGTTTAG
NANOG	F- ACAGGTGAAGACCTGGTTCC R- TTGCTATTCTTCGGCCAGTT
SOX2	F- ATGGGTTCGGTGGTCAAGT R- CTGATCATGTCCCGGAGGT

## Abbreviations

2D	Two-dimensional
3D	Three-dimensional
BSA	Bovine serum albumin
Col1a1	Collagen 1a1
ECM	Extracellular matrix
EP	Electroporation
FBS	Fetal bovine serum
GAPDH	Glyceraldehyde 3-phosphate dehydrogenase
HA	Hyaluronic acid
HAS1	Hyaluronic acid synthase 1
HFIP	Hexafluoroisopropanol
HOE	Hoechst 33,342
MFI	Mean fluorescence intensity
MMP2	Matrix Metalloproteinase 2
PBS	Phosphate buffer
PCL	Poly (є-caprolactone)
PI	Propidium iodide
SEM	Scanning electron microscopy
TCP	Tissue culture polystyrene
β-cat	β-Catenin
